# RSM-Based Modelling and Optimization of the Synergistic Effects of Waste Tyre Metal Fibre on the Electrical Resistivity and Mechanical Properties of Asphalt Mixes

**DOI:** 10.3390/polym18091042

**Published:** 2026-04-25

**Authors:** Arsalaan Khan Yousafzai, Muhammad Imran Khan, Mohamed Mubarak Abdul Wahab, Jacob Adedayo Adedeji, Xoliswa Evelyn Feikie, Nura Shehu Aliyu Yaro

**Affiliations:** 1 Department of Civil Engineering, Faculty of Civil, Agricultural & Mining Engineering, University of Engineering & Technology Peshawar, Peshawar 25120, Pakistan; 2 Department of Civil and Environmental Engineering, Universiti Teknologi PETRONAS, Seri Iskandar 32610, Malaysia; 3 Department of Civil Engineering, College of Engineering, Imam Mohammad Ibn Saud Islamic University (IMSIU), Riyadh 11432, Saudi Arabia; 4 Sustainable Environment and Transportation Research Group (SET-RG), Department of Civil Engineering Midlands, Durban University of Technology, Private Bag X01, Scottsville, Pietermaritzburg 3021, South Africa; 5 Sustainable Environment and Transportation Research Group (SET-RG), Department of Civil Engineering and Geomatics, Durban University of Technology, Durban 4000, South Africa

**Keywords:** hot mix asphalt, waste tyre metal fibre, RSM, Marshall properties, electrical resistivity, optimization

## Abstract

The disposal of waste tyres presents a significant environmental challenge, necessitating sustainable, high-value recycling solutions. This study explores the incorporation of waste tyre metal fibre (WTMF) into hot mix asphalt (HMA) to enhance mechanical performance while reducing its electrical resistivity as well as the landfill burden. The primary goal of this research is to apply response surface methodology (RSM) to experimental data for modelling and optimizing WTMF-modified HMA mixes by capturing the coupled effects of fibre reinforcement and binder content on mechanical and functional performance. The microstructural characteristics of WTMF were examined using scanning electron microscopy (SEM), energy-dispersive X-ray spectroscopy (EDS), and X-ray diffraction (XRD). WTMF-modified mixes containing five WTMF dosages (from 0% to 1.50%) and bitumen contents from 4% to 6% were prepared and tested in the laboratory. The resulting dataset was used for RSM modelling, with WTMF and bitumen contents as input factors and Marshall stability, flow, porosity, and electrical resistivity as response variables. The central composite design (CCD) technique was employed to quantify interaction effects and to identify statistically significant trends. The developed models were validated using statistical indicators, and optimal mixture compositions were determined and experimentally verified. Microstructural analysis revealed WTMF’s irregular, rough surface with microcracks and pits, aiding crack-bridging and stress transfer. RSM results indicated 0.71% WTMF and 5.1% bitumen as an optimal combination of factors. Furthermore, high R^2^ (>0.80) and adequate precision (>4.0) values from analysis of variance (ANOVA) underscore the significance of the proposed models, revealing a robust correlation between experimental and predicted data. This study demonstrated WTMF’s potential to be used in conventional HMA mixes, offering a sustainable recycling pathway for waste tyres.

## 1. Introduction

The world’s most widely utilized composite construction material for the construction of highways is asphalt [1,2]. Modifications are rapidly being made to asphalt materials to satisfy sustainability goals [3]. Pavement mixes being more resistant to damage have a prolonged service life as well as lower maintenance costs [4]. This is achieved by adding various additives in traditional asphalt mixes [5]. Researchers have increasingly used virgin additives to enhance sustainability, but these materials are often costly. Consequently, incorporating waste materials as additives provides a dual benefit: they are more affordable and contribute to environmental sustainability. The use of waste metallic additives offers several advantages like enhancing the electrical conductivity, bringing a self-sensing ability to traditional asphalt mixes that can be further used for traffic monitoring, pavement damage sensing, guidance of autonomous vehicles, non-destructing testing, snow melting and de-icing, and many other applications [6,7,8,9,10,11,12,13,14,15].

Several types of additives are used for imparting electrical conductivity into asphalt, the most commonly used ones being carbon fibre, steel fibre, aluminium fibre, steel wool [16,17], carbon nanotubes [7,18], graphene (nanometre-level) [16], graphite powder (micrometre-level), carbon black, nickel powder, iron tailings aggregates [13], copper slag [19], coke [20], metal and steel shavings, etc., [7,12,13,19,21,22,23,24]. The most typically used additive is steel fibre, as studies have reported a tensile strength of up to 502 MPa for a single steel fibre with diameters varying from 6 to 20 mm and lengths from 1 to 9 mm [8,25]. Moreover, one common form of metal additives is steel wool fibre [17]. Wang et al. [8] reportedly achieved significantly increased Marshall stability, rutting resistance, and tensile strength with steel wool fibre-modified asphalt specimens. The reason being the fact that well-distributed steel fibres make the mix capable of transferring more stress due to the formation of a complex 3D structure. Similar results were reported by Shaffie et al. [26] showing improved stability and flow results with the use of steel fibre in an asphalt mixture. Furthermore, the same study reported improvement in dynamic creep and moisture susceptibility because of adding steel fibre. However, the additives adopted in these studies are mostly virgin and increase the overall cost. On the contrary, utilization of waste materials as metallic additives can help reduce the cost involved, maintain the mechanical performance needs, and address the issue of environmental sustainability. In such conditions, waste tyres could be a viable option due to huge global production. Since global tyre production is expected to exceed 2.7 billion units by 2027 [27,28], these are considered to be one of the largest landfill wastes which pose significant environmental challenges [29]. According to Abdulrhman et al. and Rogachuk et al. [30,31], around 1.5 billion tyres are wasted after their end-of-life annually. Moreover, according to Valentini et al. [32] a passenger car tyre and a commercial truck tyre are composed of 11 to 21% by weight of steel wire alone, respectively. Alongside other reasons, this material is used to reinforce the tyre. Given these data, a safe estimate shows that more than 5 million tonnes of steel wire is discarded from these tyres annually. Hence, there is a huge amount of steel wire generated from waste tyres, thus presenting a substantial opportunity for recycling and sustainable material recovery. Therefore, it is very important to utilize such waste for reducing the overall amount of waste going into landfill sites.

A typical pattern of electrical conductivity curve can be divided into four phases: insulated phase, transition phase, conductive phase, and excess of additives phase [8,25]. The term ‘percolation/seepage threshold’ refers to the optimum amount of conductive additive which makes the asphalt exhibit the least resistivity while maintaining its required mechanical performance. The point where an abrupt insulator-to-conductor transition can be observed is the percolation threshold [33]. The threshold range is usually very narrow, so researchers usually try to transform this rapid drop into a gradual drop to achieve a wider optimum range. Further addition of a conductive additive beyond this point has no impact on increasing the conductivity as it reaches the fourth phase [8,13,14,33,34]. Hence, keeping an optimized balance between the electrical conductivity and mechanical properties of conductive asphalt due to the contents of the conductive additive is very important and a key challenge for its anticipated intelligent application to ensure long service life of the pavement [8,9,35,36]. The two-probe method, on the other hand, has been used by many researchers for measuring the electrical resistivity in asphalt specimens [5,8,12,13,16,33,34,35,36,37,38,39,40,41,42,43]. This method is observed to be the most adopted in studies, primarily due to its ease of use compared to the four-probe method. Resistance readings of the specimen measured with a multimeter are then converted to the electrical resistivity of that specific type of modified asphalt material using the Ohm’s law as given in Equations (1)–(3) [7,11,13,20,33,34,36,40,42,44,45].

(1) $$R=\frac{V}{I}$$

(2) ρ=2πsR

(3) $$\rho =\frac{R\times S}{L}$$

Here, ρ = resistivity (Ω-m); s = distance between electrodes (m); R = resistance of sample between electrodes (Ω); S = electrode–specimen contact area (m^2^); L = specimen height, same as the distance between electrodes (m); V = voltage measured between electrodes (volts); and I = constant current (amps).

Response surface methodology (RSM) is a collection of statistical techniques used for modelling and analysing the relationships between multiple input variables and a response of interest. It is primarily employed in process optimization and experimental design, where the goal is to identify the optimal conditions for a desired outcome. RSM is particularly effective when dealing with complex processes where multiple factors influence the response, and interactions or non-linear effects are expected. By fitting a mathematical model to the data, RSM helps to explore and visualize the response surface, which represents the relationship between the factors and the response. The methodology enables researchers and practitioners to systematically evaluate the effects of the factors, identify significant interactions, and optimize the process or product performance with a minimal number of experiments. This technique has been widely used in asphalt research. Memon et al. [46] applied RSM to model and optimize the mixing parameters (temperature, speed, and time) for petroleum-sludge-modified bitumen, where the optimization outcomes confirmed that RSM can reliably identify the optimal conditions under which the modified binder meets key performance criteria. Imran et al. [47] employed RSM to design and optimize the cementitious grout composition for semi-flexible pavements, effectively modelling how the water–cement ratio and superplasticiser dosage influence grout flow and compressive strength. Another study done by the same author [48] utilized RSM to design the experimental matrix and statistically analyse how replacing OPC with irradiated PET and silica fumes affects the fresh and hardened properties of cementitious grouts used in semi-flexible pavement systems. Siddiq et al. [49] employed RSM to systematically evaluate and optimize toner content, polymer content, and mixing temperature in waste-modified asphalt binders. A study done by Nouman et al. [50] applied RSM to design, analyse, and optimize cementitious grout formulations incorporating waste marble dust for semi-flexible pavements, effectively modelling how the MD content and w/c ratio influence flowability and strength. Alnadish et al. [51] in their study utilized RSM for single, tandem, and tridem axle configurations for generating stress prediction models for traffic loads on flexible pavements. Khan et al. [52] applied RSM to model and optimize the influence of nitrile butadiene rubber (from waste surgical gloves), temperature, and loading frequency on the rheological and mechanical responses of asphalt mixes. Moreover, Imran et al. [53] employed RSM to model and optimize the effects of pumice stone ash content and water–cement ratio on the flowability and compressive strength of cementitious grouts for grouted macadam pavements. The RSM-based analysis enabled the identification of optimal combinations (e.g., 17.25% PPS at 0.35 w/c) that balance workability and strength, demonstrating RSM’s effectiveness in both performance optimization and promoting sustainable binder substitution in asphalt-related applications. Also, Nasir et al. [54] employed RSM with a central composite design (CCD) to optimize coconut-fibre-modified hot mix asphalt (HMA), evaluating the effects of fibre content, fibre length, and bitumen content on Marshall stability and flow. The RSM-based optimization successfully identified a mix with improved mechanical performance and validated predictive accuracy, demonstrating RSM’s utility in reducing experimental trials while enhancing sustainable asphalt mix design.

The rising volume of waste tyres is becoming a major environmental concern, and traditional HMA is also known to have some limitations, such as the possibility of cracks and ruts and the lack of a self-sensing effect. On the other hand, waste tyre metal fibres have the advantage of waste tyre recyclability and the improvement of the properties of asphalt mixtures, both mechanically and electrically. Thus, the main aim of the present research is to develop sustainable and environmentally friendly asphalt pavements that are not only durable and have the required properties but also have the effect of sensing, addressing the challenges of waste tyres and infrastructure performance.

Despite the extensive research on polymer- and fibre-modified asphalt mixtures, several important issues still need to be addressed. There is a lack of investigation into using waste tyre metal fibres as a functional additive in HMA mix, and there is no clear understanding of the relationship between the quantitative values of various characteristics of fibres and the macroscopic properties of HMA mix (such as Marshall stability and flow values, volumetric properties, and electrical resistivity). There is also a lack of multi-objective optimization of HMA mix using various properties of waste tyre metal fibres and bitumen. Hence, it is important to address these gaps and provide proper guidance on using this sustainable pavement construction material.

## 2. Significance and Scope of This Study

**Figure 1:**
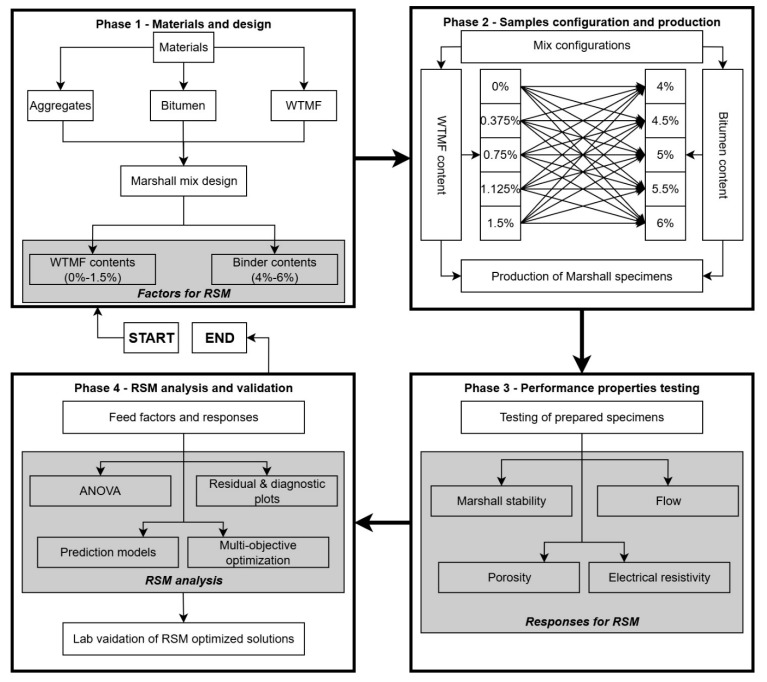
Experimental program and scope of the research.

The utilization of waste tyres due to their huge scale of production and being one of the biggest types of landfill waste is a challenge. Moreover, production of innovative asphalt material is also needed to cater to sustainable development goals and higher traffic load requirements. While commercially manufactured fibres have shown promising mechanical improvements in asphalt mixtures, their cost and sustainability concerns have motivated recent efforts toward alternative and recycled fibre systems [55]. Hence, this study focuses on utilizing waste tyre metal fibre (WTMF) as an additive to modify conventional asphalt mixes to assess the mechanical performance of such modified mixes in the laboratory. Mechanical performance is a key parameter that is supposed to be assessed before its field implementation. This laboratory study investigates the effects of using WTMF in various proportions, i.e., 0% (control), 0.375% (series A), 0.75% (series B), 1.125% (series C), and 1.5% (series D), as an additive, along with varying optimum contents of bitumen determined for each mix series. The selection of experimental parameters and WTMF content levels in this study was guided by a comprehensive review of previous research on fibre-modified asphalt mixtures. These dosage levels were further validated through preliminary trial mixing to ensure uniform fibre distribution. Next, RSM is employed on the data obtained from the laboratory experiments, which contain WTMF and bitumen contents as factors for the analysis and the Marshall stability, flow, electrical resistivity, and porosity as the responses. Statistical analyses were performed and empirical models were developed; multi-objective optimization was performed as well to obtain the optimized solution for the given criteria. Accordingly, the novelty of this study lies in integrating laboratory characterization of WTMF-modified asphalt with RSM-based modelling and multi-objective optimization, providing statistically validated guidance for a sustainable mix design using recycled-tyre-derived metal fibres. Figure 1 outlines the overall scheme and scope of this study. The subsequent sections provide details about the materials and experimental program, results and discussion, and finally the conclusions drawn.

## 3. Materials and Methodology

### 3.1. Materials

**Figure 2:**
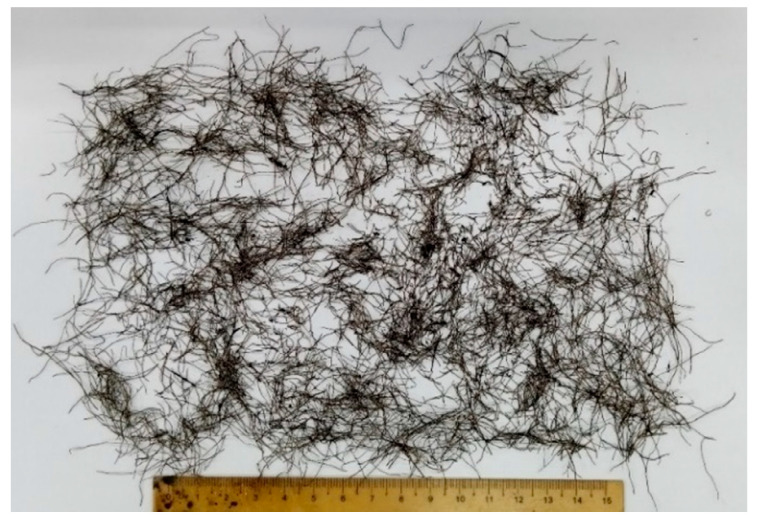
Waste tyre metal fibre used in this research.

**Figure 3:**
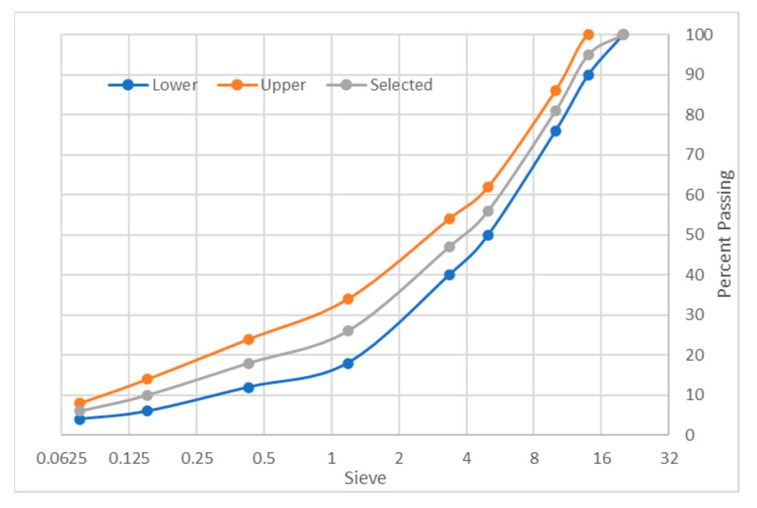
JKR’s aggregate gradation limits.

This research was carried out using local highway construction materials available in Perak, Malaysia. Aggregates were acquired from Sunway Quarry Industries. Bitumen with a penetration grade of 60/70 was acquired from KBC Refinery at Terengganu, keeping in mind its current usage in Malaysia. Table 1 shows the physical properties of the aggregates and bitumen used in this study. WTMF was used as the primary additive, as shown in Figure 2, which was firstly extracted in raw form from waste tyres. Then, it was cleaned of any unwanted rubber particles and then batched according to the required contents. The content of WTMF is selected such as to conform to previous research studies, as well as to address the issue of accumulation, heterogeneous mixing, and inadequate compaction at higher contents. Moreover, Malaysia’s JKR specifications were adopted for the Marshall mix design [56]. Asphaltic mix having a maximum nominal aggregate size of 14 mm (i.e., AC-14) was adopted for aggregate gradation, representing the wearing course of the pavement. Accordingly, the aggregates were sieved through the required sieve sizes for achieving the required combinations based on particle size. Figure 3 shows the aggregates’ gradation limits utilized in this research.

Field Emission Scanning Electron Microscopy (FESEM) was utilized to examine the surface morphology of WTMF. FESEM is commonly employed to analyse the morphology of modified asphalt mixes, providing precise frequency histograms that display the shape, size, and distribution of ingredients [5,19,57]. The surface morphology of the additives used was analysed using a Carl-Zeiss ultra-high-resolution SUPRA 55VP FESEM (Oberkochen, Germany). To minimize the structural changes caused by electron beam heating during scanning, a cryogenic step was employed. The samples, both non-irradiated and gamma-irradiated, were scanned at a temperature of −260 °C and a pressure of 30 Pa. The FESEM equipment operated at 200 kV, and to enhance sample conductivity, the WTMF samples were prepared as slides and coated with an ultra-thin gold film. Moreover, X-ray diffraction (XRD) analysis was conducted to investigate the crystallization structure of WTMF, providing insights into the relative proportions of mineral constituents and crystalline phases—regular 3D arrangements of atoms in a solid [58,59]. It is a non-destructive technique that yields precise information on the crystallographic structure, chemical composition, and physical properties of materials. Following the ASTM D3906 standard [60], the XRD test was carried out on WTMF samples using a Malvern PANalytical X-ray diffractometer, model Xpert^3^ Powder (Almelo, The Netherlands).

### 3.2. Sample Preparation

**Figure 4:**
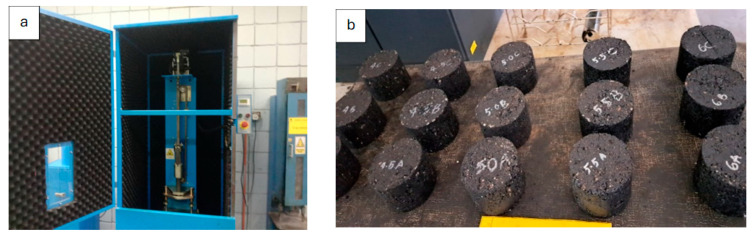
(**a**) Marshall compactor used in preparation of samples. (**b**) Prepared Marshall specimens.

The initial stage was to determine the optimum binder content (OBC) for the control samples as well as each modified mixture type with the specific content of WTMF. The Marshall mix design procedure was adopted for determining the OBC, whose objective is to optimize the quantity of binder needed for a specific mix type to make a durable mix. The OBC was determined for the control as well as WTMF-modified mixes in accordance with the technique established by the Asphalt Institute [61]. A range of 4 to 6% bitumen was selected with an increment of 0.5%. The parameters taken into consideration for determining the OBC were stability, flow, voids in mineral aggregate (VMA), porosity, bulk specific gravity (G_mb_), and voids filled with binder (VFB). In the first phase, various proportions of bitumen (4%, 4.5%, 5%, 5.5%, and 6%) were used to calculate the OBC for each mix series (control and series A–D). It is noteworthy that the OBC for each type of mix was different from the other because of varying contents of WTMF in each mix. Marshall specimens were produced in accordance with ASTM D6926-20 [62], for which 1200 g of graded aggregates was added to the determined amount of bitumen and the selected amount of WTMF additive for preparation of each Marshall specimen. The aggregate composition for coarse and fine aggregates and mineral filler was 44%, 50%, and 6%, respectively. These were firstly placed in oven at ~150 °C for an ample period to completely remove any moisture contained in them. The dry mixing process was adopted for blending the aggregates with binder added at pre-calculated OBC values. During mixing, additives were added in intervals of small quantities. Next, the mix was shifted to pre-heated moulds having approximately 101.6 mm diameter and 63.5 mm height for maintaining the same mixing temperature. Seventy-five blows were given to each sample per face using a standard Marshall compactor (Model UTAS-0683E, UTEST, Ankara, Turkey) dropped from a standard height of 457 ± 3 mm, as shown in Figure 4a. Seventy-five Marshall samples were prepared for the determination of Marshall parameters and OBC of both the control and modified mixes. Figure 4b shows the samples prepared and left overnight to allow them to cool down to room temperature. It is noteworthy that each test was conducted on a minimum of three individually prepared and tested specimens per mix series, resulting in an extensive experimental dataset while maintaining practical feasibility.

### 3.3. Experimental Methods

**Figure 5:**
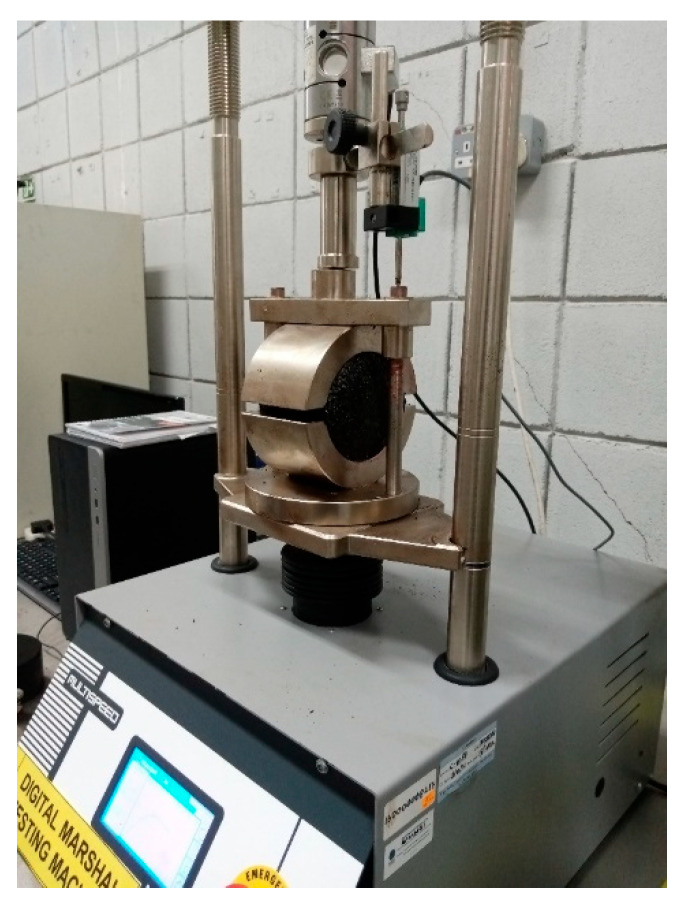
Marshall testing machine used in this study.

Stability and flow are essential tests for assessing the bituminous mixture’s resistance to deformation and exposure to traffic load. The former represents the mix’s tensile strength and its ability to resist rutting at high temperatures, whereas the latter represents the mix’s rutting resistance in terms of permanent strain which occurs at failure during Marshall testing. Dry weight, weight in water, and saturated surface dry (SSD) weight were first determined for calculating the volumetric properties. After which, the testing was done in accordance with ASTM D6927 [63]. The Marshall testing equipment used in this study is shown in Figure 5. The specimens were conditioned in a water bath at 60 °C for 25–30 min for simulating service temperatures. A rate of load application was maintained at 50.8 mm/min while making sure that the jig’s temperature was maintained at 60 °C. The peak load at failure is noted as a value of stability, whereas the flow value is obtained through the flowmeter installed in the equipment.

**Figure 6:**
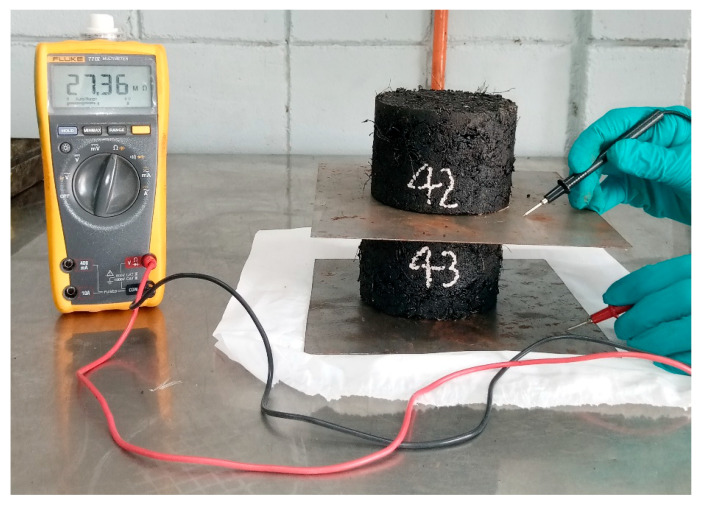
Electrical resistivity determination.

The purpose of finding the electrical resistivity of asphalt Marshall specimens is to determine the optimum content for reaching the percolation threshold where a sudden drop in the resistivity is observed without impairing its mechanical properties. The two-probe method was used to determine the electrical resistivity of the asphalt mixes at room temperature, as shown in Figure 6. Copper plates having dimensions of 200 × 200 mm were used as electrodes, and the resistance of Marshall samples was measured using the modern and reliable Fluke 77 IV multimeter (Fluke Corporation, Everett, Washington, DC, USA) having a resistance measurement capacity of 500 MΩ. A Marshall sample was placed above the copper plate to apply an ample load, thereby maximizing the contact area between the specimen surface and the electrode and minimizing contact resistance at the interface. This loading approach is commonly adopted in two-probe measurements to stabilize electrical contact and improve the repeatability of conductivity readings. Since the resistance is dependent on the sample’s physical dimensions, this value is converted into resistivity using Equations (1)–(3) to make it independent of the specific size of the sample, and the value could be considered for the whole specific asphalt mix.

### 3.4. Application of RSM

#### Design of Experiments

**Figure 7:**
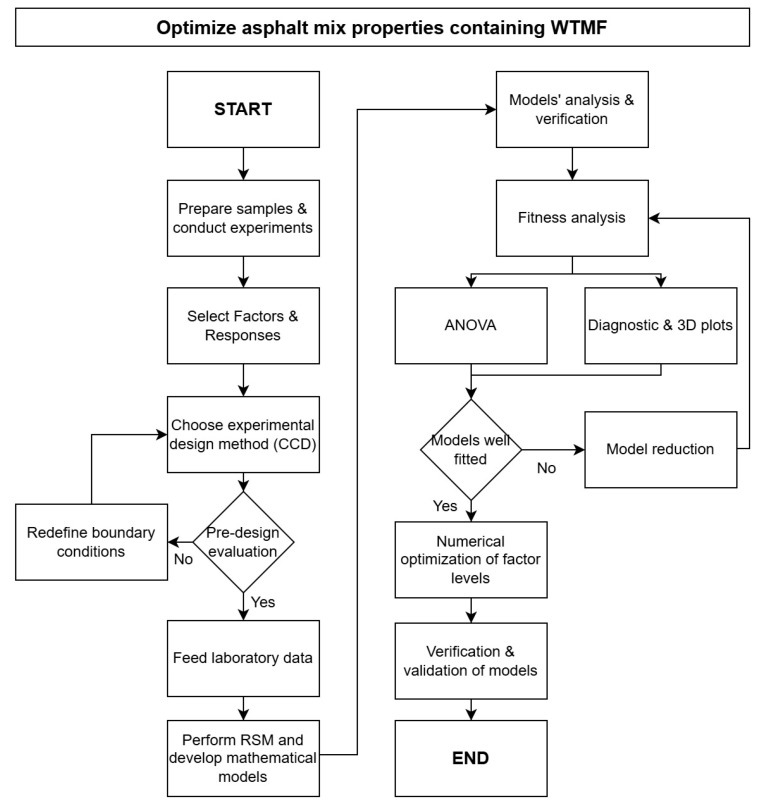
Flowchart of the RSM process.

In the current study, Design Expert^®^ version 13 software was used to design the experiments. The central composite design (CCD) approach was used for the design of experiments (DOE) in the current study. It is a widely and generally used experimental design in RSM for modelling and optimizing processes. CCD is particularly effective in exploring the relationships between multiple factors and determining the optimal conditions for a desired response. It is designed to estimate both the linear and quadratic effects of the factors, as well as interactions, with relatively few experimental runs compared to a full factorial design. CCD is particularly useful when the primary objective is to identify optimal conditions for a response, such as in process optimization or product development, where understanding the impact of various factors is crucial. The detailed flowchart for understanding the RSM process is shown in Figure 7.

WTMF and bitumen contents were considered as factors or independent variables, whereas electrical resistivity, Marshall stability, flow, and porosity were considered as responses or dependent variables. These variables, along with their corresponding units and codes, are summarized in Table 2. The responses were calculated by quadratic and cubic models; hence, second-degree and third-degree polynomial equations (Equations (4) and (5)) were used for calculating the responses. The quadratic model is the most commonly used in RSM as it can account for both linear and non-linear effects, whereas the cubic model is a more complex form that includes cubic terms to account for higher-order non-linear effects. The experimental factors and their coded levels with upper and lower limits for RSM are shown in Table 3.

(4) $$Y=\beta_{0}+\sum_{i=1}^{k}\beta_{i}X_{i}+\sum_{i=1}^{k}\beta_{ii}X_{i}^{2}+\sum_{i=1}^{k}\sum_{j=i+1}^{k}\beta_{ij}X_{i}X_{j}+\epsilon$$

(5) $$Y=\beta_{0}+\sum_{i=1}^{k}\beta_{i}X_{i}+\sum_{i=1}^{k}\beta_{ii}X_{i}^{2}+\sum_{i=1}^{k}\sum_{j=i+1}^{k}\beta_{ij}X_{i}X_{j}+\sum_{i=1}^{k}\beta_{iii}X_{i}^{3}+\sum_{i=1}^{k}\sum_{j=i+1}^{k}\beta_{iijj}X_{i}^{2}X_{j}^{2}+\epsilon$$

In the above equations, Y is the response variable, $\beta_{0}$ is the intercept term, $\beta_{i}$ is the linear coefficient (effect of each factor), $\beta_{ii}$ is the quadratic coefficient (effect of the squared terms of each factor), $\beta_{ij}$ is the interaction coefficient (effect of the interaction between pairs of factors), $\beta_{iii}$ is the cubic coefficient (effect of the cubic terms of each factor), $\beta_{iijj}$ is the interaction coefficient for the squared terms of different factors, $X_{i}$ and $X_{j}$ are the independent variables, and ϵ is the error term (residuals), if any.

## 4. Results and Discussion

### 4.1. WTMF Characterization

#### 4.1.1. Scanning Electron Microscopy

**Figure 8:**
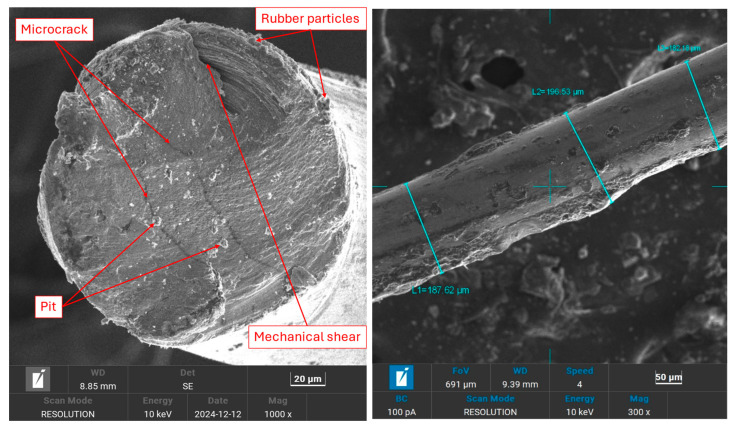
SEM images of WTMF: (**left**) diametrical face; (**right**) longitudinal face.

The microstructural and compositional analysis of WTMF was conducted using Scanning Electron Microscopy (SEM) coupled with energy-dispersive spectroscopy (EDS). Regarding the microstructural analysis, Figure 8 (left) presents the SEM image of the diametrical face of WTMF at 1000× magnification, revealing a rough and irregular surface morphology. The fibre surface exhibits microcracks and pits, which may be attributed to mechanical wear, material degradation, or exposure during the tyre shredding and extraction process. The presence of microcracks suggests that the fibre may have undergone prior stress or thermal effects, which could affect its mechanical performance when embedded in concrete. Additionally, the pitted surface texture may enhance the mechanical interlocking between the fibre and the asphalt mastic, potentially improving its pull-out resistance. However, excessive roughness or deep pits could also lead to stress concentration points, impacting the fibre’s durability under load. These characteristics may influence the fibre’s mechanical interlocking with the surrounding asphalt mastic, affecting its load transfer efficiency and durability.

The presence of microcracks and a pitted surface texture enhance mechanical interlocking between the fibre and the surrounding asphalt mastic, which can improve adhesion and load transfer efficiency under applied stresses. However, excessive surface roughness or deep pits may also act as stress concentration sites, potentially affecting fibre durability under repeated loading. In addition, the surface condition of WTMF, including the presence of oxide layers as indicated by EDS analysis, may influence electrical behaviour by increasing contact resistance between fibres and the asphalt matrix. Consequently, the electrical conductivity of WTMF-modified mixtures depends not only on fibre content and distribution but also on surface condition and inter-fibre contact quality.

Figure 8 (right) illustrates the longitudinal face of WTMF at 300× magnification, where three diameter measurements were taken to assess its dimensional consistency. The recorded diameters were 187.62 μm, 196.53 μm, and 182.18 μm, resulting in an average diameter of 188.78 μm. The slight variation in diameter suggests that the fibre exhibits non-uniformity, which could be attributed to manufacturing inconsistencies or surface wear. This variation may influence its dispersion in the asphalt mastic and the efficiency of load transfer when subjected to stress.

The aspect (i.e., length-to-diameter) ratio is crucial in determining the reinforcing effectiveness of the fibre. It was based on the fibre length which varies between 3 mm and 9 mm, so an average length of 6 mm (6000 μm) was considered for calculating the aspect ratio. With an average diameter of 188.78 μm, the aspect ratio was determined to be approximately 32. This high aspect ratio indicates that the fibres are long and thin, which is desirable in an asphalt mix to reinforce its structure and improve the interfacial bonding. Such a high value of aspect ratio generally enhances the mechanical interlocking and crack-bridging ability of the fibre in concrete, contributing to improved toughness and durability.

**Figure 9:**
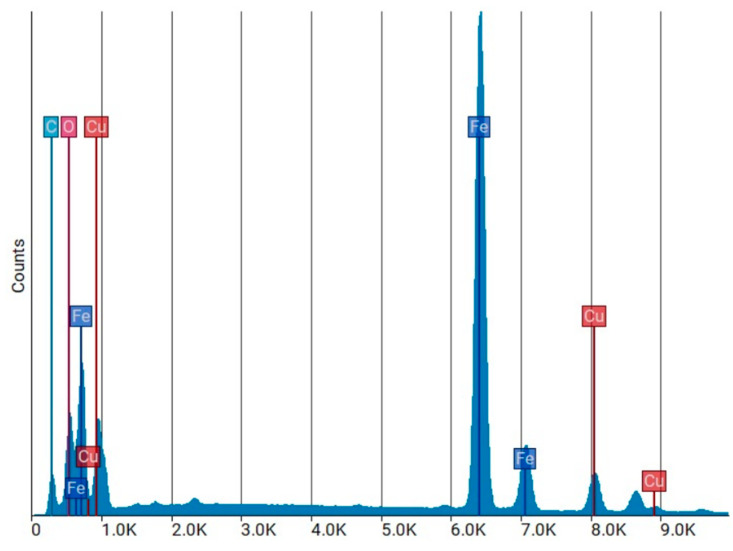
EDS elemental spectrum.

The EDS elemental spectrum (Figure 9) provides a graphical representation of the elements detected in the WTMF sample. The *X*-axis represents the energy levels (in kiloelectron volts, keV) of the emitted X-rays, while the *Y*-axis denotes the intensity (counts) of detected X-rays at each energy level. Each element in the sample produces characteristic X-ray peaks at specific energy levels, allowing for elemental identification. The position of a peak on the *X*-axis corresponds to the element’s unique energy signature, while the peak height on the *Y*-axis indicates the relative abundance of that element in the scanned area. Higher peaks suggest a greater presence of the corresponding element. The quantitative analysis of the detected elements is summarized in Table 4, which includes their atomic and weight fractions, respectively.

The results above indicate that iron (Fe) is the dominant element, with a weight percentage of 51.65%, confirming the metallic nature of the fibre. The presence of oxygen (12.13% Wt.%) suggests surface oxidation, likely due to environmental exposure or corrosion. Carbon (C) is present in a significant amount (57.32% At.%), which may be attributed to the residual rubber, coatings, or polymeric components of the original tyre reinforcement. The SEM and EDS findings confirm that WTMF primarily consists of iron, with additional contributions from carbon and oxygen. The presence of an oxide layer could influence the bonding efficiency of the fibre within cementitious materials, while the high carbon content suggests remnants of the original rubber or polymer coatings. These factors are crucial in understanding the performance and durability of WTMF as a reinforcement material in asphalt modification.

#### 4.1.2. X-Ray Diffraction

The X-ray diffraction (XRD) analysis covers key aspects of the WTMF samples. The peak parameters analysis identified peak positions, intensities, and full width at half maximum (FWHM) values to understand the phase presence and broadening effects. Crystallite size analysis used Scherrer’s equation to estimate the crystallite sizes, indicating nanoscale grain size and potential strain effects. The XRD analysis was conducted with Cu anode material and Kα radiation wavelength type with λ = 1.5406 Å. The scan was performed over a 5–90° 2θ scan range and a step size of 0.0263°. The obtained diffraction pattern was analysed using PANalytical Data Viewer 1.9a software, and phase identification was carried out using reference data from the International Centre for Diffraction Data (ICDD) database [64].

**Figure 10:**
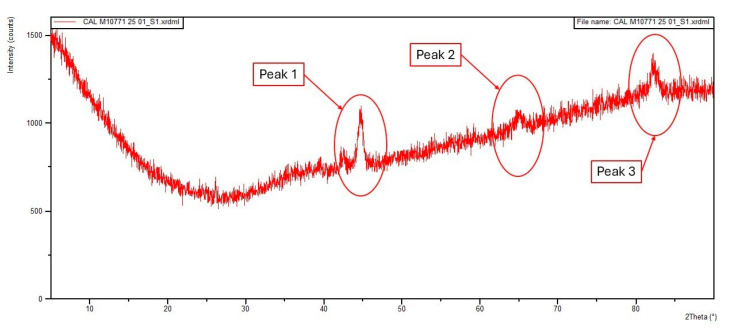
XRD pattern of WTMF sample.

Determination of the crystalline phases present in the WTMF sample was done. As shown in Figure 10, the obtained diffraction pattern revealed three major peaks at 44.785°, 65.689°, and 82.261° (2θ). These peak positions were compared with standard diffraction data from the ICDD database. The results indicate that the peaks closely correspond to those of body-centred cubic (BCC) iron (α-iron or ferrite), which confirms that the dominant phase in the WTMF sample is BCC iron. Additionally, energy-dispersive spectroscopy (EDS) from the FESEM analysis confirmed that iron (Fe) and carbon (C) are the major elements in the sample. However, the insignificant peak near 26.6° in the XRD pattern suggests that graphitic carbon is present in negligible amounts.

Moreover, the XRD peak parameters analysis was done to further analyse the diffraction pattern. The peak parameters, including position (2θ), net height (counts), and full width at half maximum (FWHM), were evaluated using software. The results are summarized in Table 5. The peak broadening observed in the FWHM values may be indicative of micro-strain, crystallite size effects, or instrumental broadening. The peak with the largest FWHM (1.337°) at 82.261° position suggests a possible reduction in crystallite size or increased strain effects in this reflection.

Once the peak parameters were obtained, the crystallite sizes were estimated using Scherrer’s equation (Equation (6)) to get insights into the microstructural properties of the material.

(6) $$D=\frac{K\lambda }{\beta cos\theta }$$

In the above equation, D is the crystallite size (nm), K is the shape factor (taken as 0.9), λ is the X-ray wavelength (1.5406 Å for Cu Kα radiation), β is the full width at half maximum (FWHM) in radians, and θ is the Bragg angle. The calculated crystallite sizes for each peak are shown in Table 6.

The results indicate that the crystallite sizes range between 78.9 nm and 113.65 nm, where peak 2 (65.689°) has the largest crystallite size (113.65 nm), indicating a more crystalline structure for this phase, and peak 3 (82.261°) has the smallest crystallite size (78.89 nm), possibly due to strain or smaller grains in this phase.

While the microstructural features of WTMF, such as fibre distribution and surface characteristics, were observed, their direct correlation with macroscopic mechanical performance parameters was not assessed in this study. These microstructural characteristics of WTMF were considered in the design of experiments and the optimization process using RSM, and their influence on the selected response variables is reflected in the predicted optimum mixture parameters.

### 4.2. Marshall and Volumetric Properties

The Marshall parameters are one of the most widely used and important aspects of pavement performance, since they provide critical insights into the performance of asphalt mixes. Table 7 illustrates the results for Marshall parameters and volumetric properties for the control and WTMF-modified mixtures.

**Figure 11:**
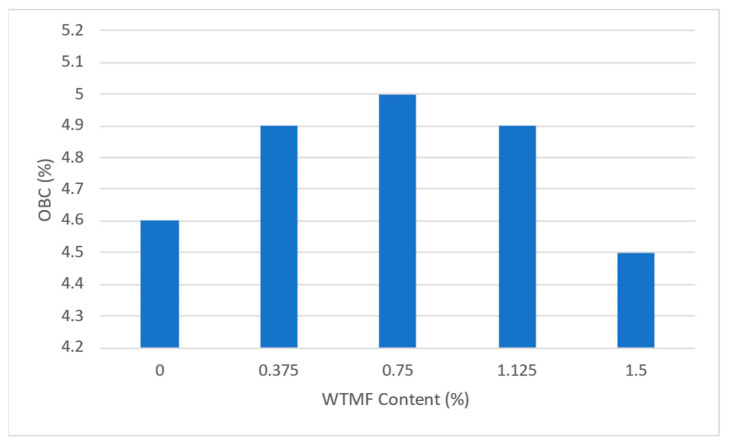
Effect of WTMF on OBC.

**Figure 12:**
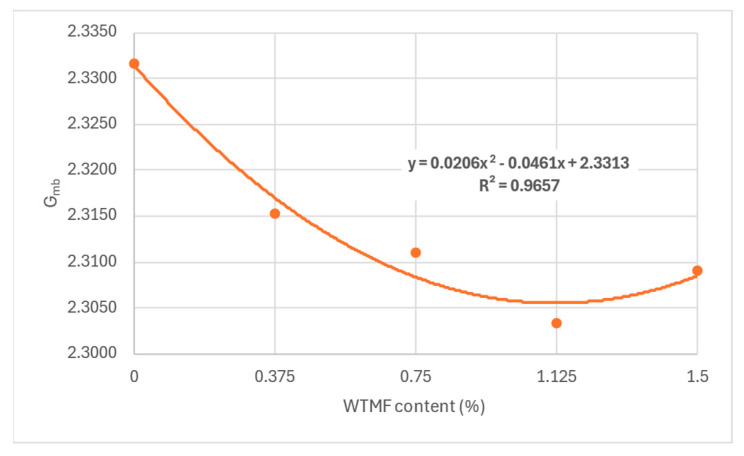
Effect of WTMF content on bulk specific gravity.

**Figure 13:**
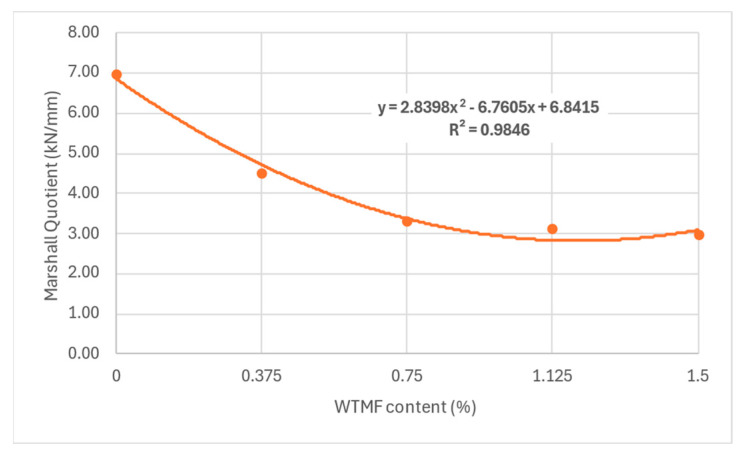
Effect of WTMF on Marshall Quotient.

**Figure 14:**
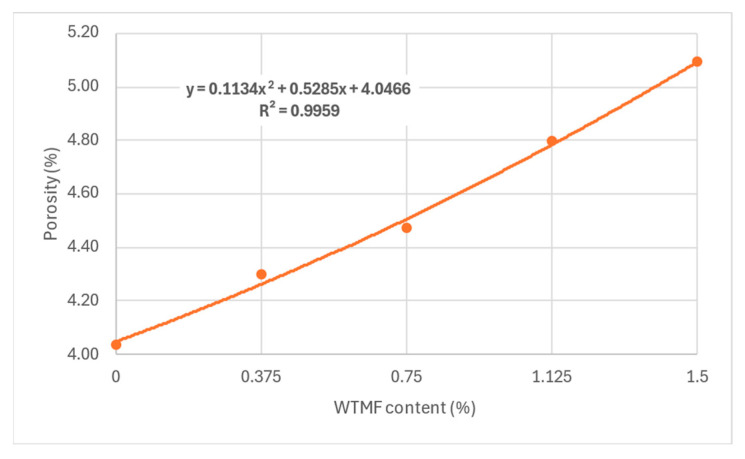
Effect of fibre content on porosity.

**Figure 15:**
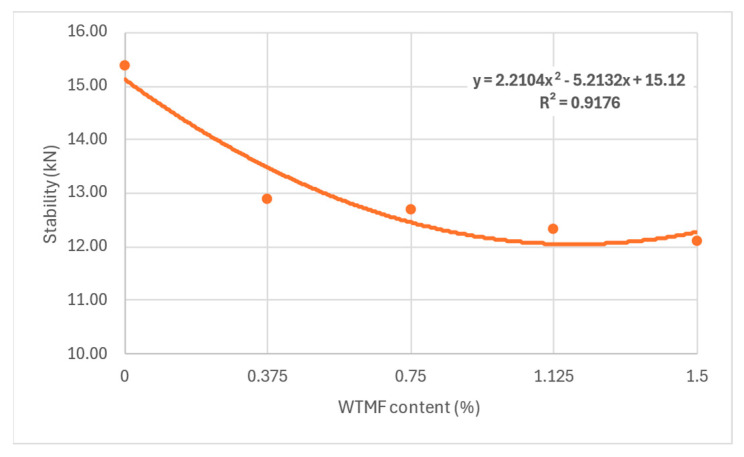
Effect of WTMF content on Marshall stability.

**Figure 16:**
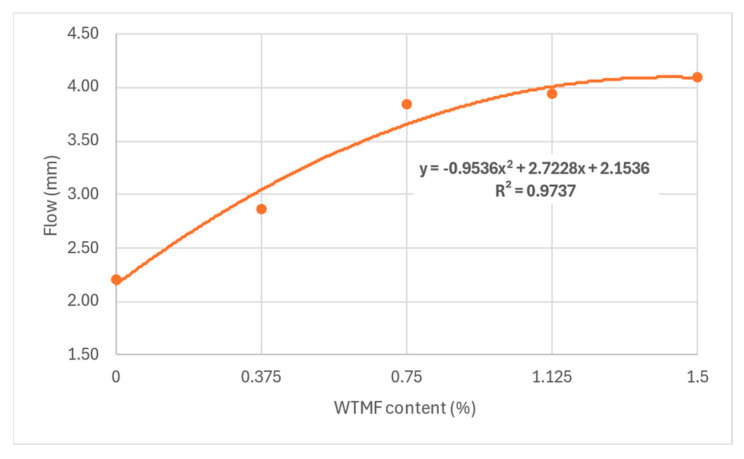
Relationship of WTMF content with flow.

From Table 7 above and Figure 11, it can be observed that the OBC varied for each mix type due to varying proportions of WTMF. The highest OBC was found for the series B mix, having a WTMF content of 0.750%. Figure 12 represents the effect of fibre content on the bulk specific gravity of all the mix types, which shows a declining effect with the increase in WTMF content. Moreover, a decline in the trend of the Marshall Quotient (MQ) was also observed with the addition of WTMF in the mixes, as shown in Figure 13. The figure represents an inverse relationship between stability and flow, with values ranging from 6.96 kN/mm for control samples to 2.95 kN/mm for the maximum WTMF content. Since it is a parameter used to indicate the resistance of an asphalt mix’s resistance to deformation, this means that the WTMF-modified mixtures are less rigid compared to the control mixture, which might be due to lower amount of internal friction in mixtures containing WTMF. Moreover, porosity was observed to increase linearly with the increase in WTMF content, as presented in Figure 14. This increase is due to a conglomeration of fibre during the mixing and compaction process. Moreover, the overall surface area of the asphalt mastic increases with the incorporation of fibre, due to which it is logical to see an increase in the porosity of the mixes with the increase in fibre content. This leads to a less dense mix, affecting the stability of the mix. Figure 15 shows the effect of WTMF content on stability, showing a decline in stability with the increase in fibre content, further supporting the relationship observed in case of air voids and fibre content. Similarly, Figure 16 shows the effect of WTMF content on the flow of asphalt mixes, showing a trend of an increase in flow with the increase in fibre content. Initially, the slope is steeper, and then the slope becomes stabilized with the increase in fibre content. Similar results were reported by Al-Bdairi et al. [65].

**Figure 17:**
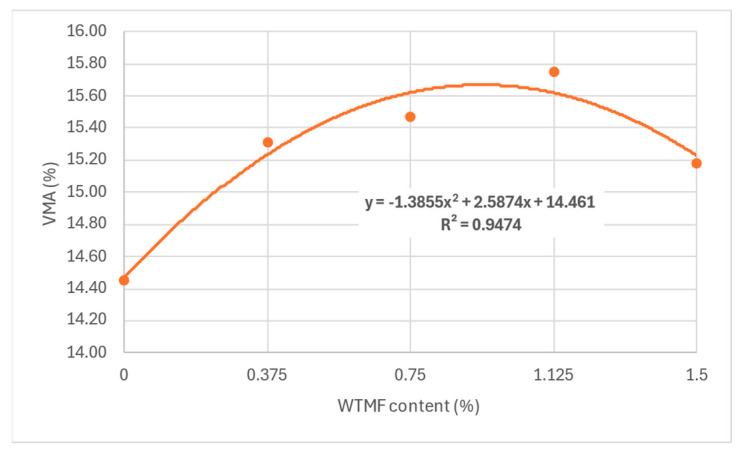
Effect of WTMF content on VMA.

**Figure 18:**
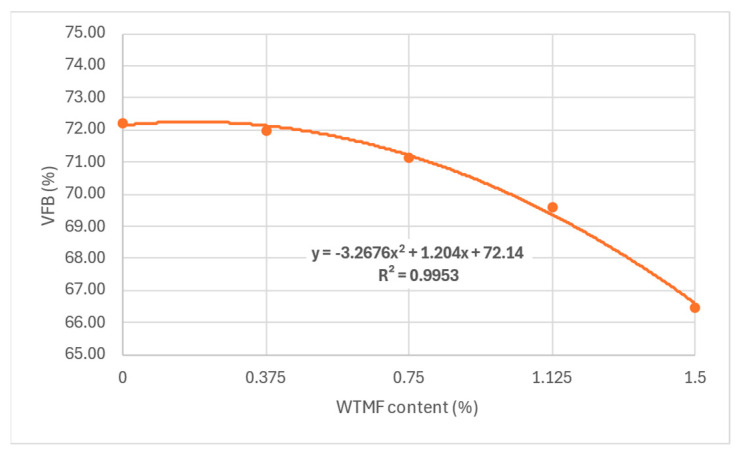
Effect of fibre content on VFB.

The maximum value of VMA was observed for content in between series B and series C, as shown in Figure 17. It can also be observed that VMA has a direct impact on the OBC, since more bitumen can be accommodated in the mix with more VMA. However, for series D, a decline in OBC also resulted in lower VMA, as shown in Figure 11 above. Similarly, as depicted in Figure 18, the VMA reduced from 72% to 66.5% with the addition of fibre, due to the absorption of bitumen by the increased fibre content’s surface area. The same argument justifies the reduction in OBC for the series D mixture.

### 4.3. Electrical Resistivity

**Figure 19:**
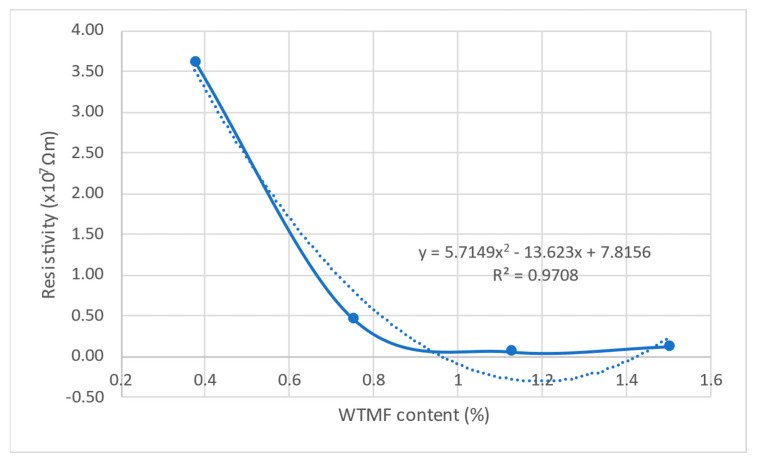
Electrical resistivity plot along with fitted trendline for the specified fibre contents in the asphalt mixes.

Electrical resistivity is a key parameter in evaluating the conductivity characteristics of asphalt mixtures, particularly when modified with conductive additives like waste tyre metal fibre (WTMF). The ability of asphalt mixtures to conduct electricity is essential for various self-sensing applications. The two-probe method was adopted since it is considered to be the most widely used method for the electrical resistivity measurement in the literature. Table 8 presents the electrical resistivity values recorded for each mix series with varying WTMF content, while Figure 19 illustrates the overall trend of resistivity with increasing fibre content.

From the results, it is evident that the control mix (without WTMF) exhibits infinite resistivity, indicating a non-conductive nature. However, with the incorporation of WTMF, the resistivity drops significantly, demonstrating the material’s transition from an insulating to a more conductive phase. The graph in Figure 19 shows a sharp decline in resistivity up to a certain fibre content, which marks the conductive region’s percolation threshold. This suggests that critical WTMF content is required to establish a continuous conductive network within the asphalt mastic. Beyond this point, the decline in resistivity slows down, indicating the excess additive zone, where additional WTMF does not significantly enhance conductivity further.

Among the modified mixtures, series A (0.375% WTMF) exhibits the highest resistivity among the fibre-modified samples, indicating that at this lower fibre content, the conductive pathways are not well established. As the fibre content increases, series B (0.75% WTMF) shows a drastic drop in resistivity, demonstrating that at this dosage, a significant conductive network begins to form. The lowest resistivity value of 6.18 × 10^5^ Ωm is observed in series C (1.125% WTMF), suggesting that this fibre content provides the optimal level of conductivity within the asphalt mix. However, in series D (1.5% WTMF), a slight increase in resistivity is recorded compared to series C. This unexpected rise in resistivity at higher fibre contents may be attributed to excessive fibre leading to agglomeration, which disrupts the continuity of the conductive network rather than enhancing it. When the WTMF content reaches 1.5%, the fibres clumping together disrupts the uniform dispersion within the asphalt mastic and severs the conductive paths, thereby raising the resistivity due to a lack of uniform fibre-to-fibre interactions. To minimize this agglomeration, the method of preparing the mixture might be adjusted. Moreover, incrementally introducing fibres into the dry mixing process, extending the mixing time, and regulating the temperature to maintain lower viscosity of the binder to facilitate easier dispersal, may also be adopted. Thus, the rise in electrical resistivity upon increasing the dosage of WTMF is not merely related to the material, since the electrical resistivity also shows more dependence on the processing conditions.

To further investigate the relationship between WTMF content and the electrical resistivity of the asphalt mixtures, a second-order polynomial trend model was developed. The resulting equation is expressed in Equation (7) with a coefficient of determination (R^2^) of 0.9708, indicating an excellent goodness of fit. This high R^2^ value confirms that more than 97% of the variation in electrical resistivity (Y) can be explained by the change in WTMF content (X), highlighting a strong functional relationship between the two parameters.

(7) $$Y=6\times 10^{7}X^{2}-1\times 10^{8}X+8\times 10^{7}$$

The electrical resistivity measurements confirm that increasing the WTMF content enhances the conductive properties of the asphalt mixes up to an optimal level, beyond which additional fibre content does not yield significant improvements. The findings highlight the importance of identifying the percolation threshold to achieve the best balance between conductivity and mechanical performance in WTMF-modified asphalt.

### 4.4. RSM Analysis

RSM was utilized for the design and analysis of several WTMF-modified mixes. Statistical analysis was performed using analysis of variance (ANOVA) to see the effect of different contents of independent parameters on the dependent parameters. The outcomes of ANOVA were then optimized using the CCD approach in RSM. The independent parameters, also known as factors, were fixed as WTMF and binder contents, whereas the dependent parameters, also known as responses, were the electrical resistivity, Marshall stability, flow, and porosity of the mixes.

#### 4.4.1. ANOVA Validation

ANOVA was utilized to estimate the significance of the independent variables (factors) and their interactions with each other. Table 9 shows the details of the models proposed for the electrical resistivity, Marshall stability, flow and porosity of the mixes based on WTMF and binder content percentages. The type of model for each response is suggested based on R^2^ values. Moreover, the models will be considered significant if the *p*-value is found to be less than 0.05.

The table above presents the ANOVA and model validation in terms of the response variables. Regarding model significance, the F-value represents the ratio of the model variance to the residual variance. A high F-value indicates that the model terms contribute significantly to the response. In this study, the F-values for all responses are relatively high, with resistivity having the highest F-value (326.51), followed by flow (45.31), porosity (30.16), and stability (23.10). The *p*-values for all models are less than 0.0001, indicating that the models are statistically significant. A *p*-value below 0.05 confirms that there is a less than 5% probability that the results occurred due to random variation, meaning the models effectively describe the relationship between the input factors and responses [66]. Moreover, the R^2^ value measures how well the model explains the variability in the response. In this study, the R^2^ values are above 0.80 for all responses, with resistivity showing the highest R^2^ (0.96), indicating an excellent fit. Stability (0.80), flow (0.81), and porosity (0.84) also show good model performance. The R^2^ of ≥0.80 shows that the model is well fitted and indicates a good agreement between the actual and predicted responses [67,68]. The adjusted R^2^ accounts for the number of predictors in the model and adjusts R^2^ accordingly. It prevents overestimation when additional non-significant terms are included. The values remain high, confirming that the models are reliable. The predicted R^2^ evaluates the model’s predictive capability. A value close to adjusted R^2^ suggests that the model can generalize new data well. Here, the values are slightly lower than adjusted R^2^ but remain high, confirming that the models have good predictive power. Adequate precision measures the signal-to-noise ratio. A value greater than four indicates an adequate model. The values obtained for all responses (ranging from 22.45 to 43.60) confirm that the models have a strong signal and can be used for predictive purposes.

Based on the model fit and significance, different models were selected for each response. Quadratic models were found to be suitable for resistivity and flow responses, suggesting that interactions between input factors are significant but higher-order interactions are not necessary. Cubic models were chosen for stability and porosity, indicating that additional curvature in the response surface improves model accuracy. Moreover, the findings confirm that RSM effectively models and optimizes the selected responses. The statistical significance of the models suggests that the input parameters (e.g., WTMF content, bitumen content, and other mix variables) have a substantial impact on resistivity, stability, flow, and porosity.

#### 4.4.2. Prediction Equations

Statistical equations were developed using RSM for predicting the responses in terms of the dependent variables. The intercept ($\beta_{0}$), linear, quadratic, and cubic coefficients ($\beta_{i},\;\beta_{ii},\;\beta_{iii}$) and the interaction coefficients ($\beta_{ij},\beta_{iijj}$), given in the generalized equations (Equations (4) and (5)), are replaced by actual factors to develop model equations for each response variable as given below (Equations (8)–(11)).

(8) $$Resistivity=+7.83\times 10^{6}-3.788\times 10^{7}X_{1}-9418.08X_{2}-4.410\times 10^{5}X_{1}X_{2}+3.213\times 10^{7}X_{1}^{2}+5.166\times 10^{5}X_{2}^{2}$$

(9) $$Stability=+11.01-0.3769X_{1}-0.2853X_{2}-0.0759X_{1}X_{2}+3.13X_{1}^{2}+1.15X_{2}^{2}-0.1281X_{1}^{2}X_{2}-3.33X_{1}X_{2}^{2}-2.46X_{1}^{3}-0.902X_{2}^{3}$$

(10) $$Flow=+3.51+0.9708X_{1}+0.4143X_{2}+0.0463X_{1}X_{2}-0.01255X_{1}^{2}+0.027X_{2}^{2}$$

(11) $$Porosity=+4.67+0.3023X_{1}-1.37X_{2}+0.8067X_{1}X_{2}-1.34X_{1}^{2}-0.0272X_{2}^{2}-0.2722X_{1}^{2}X_{2}+1.03X_{1}X_{2}^{2}+1.01X_{1}^{3}-0.3755X_{2}^{3}$$

It may be noted that the terms $X_{1}$ and $X_{2}$ are the independent variables, i.e., WTMF and binder contents, respectively. Moreover, quadratic models were suitable for resistivity and flow, indicating that their behaviour could be adequately described with second-order polynomial equations, whereas cubic models were necessary for stability and porosity, showing more complex relationships requiring higher-order terms. The models provide optimized mix proportions for achieving desired mechanical and durability properties in asphalt mixtures. It should be noted that the developed equations are empirical in nature and are valid within the investigated experimental domain, serving as predictive and optimization tools rather than mechanistic models. The significance of the models, as confirmed by ANOVA, ensures their reliability for predicting and optimizing asphalt performance under varying mix designs.

#### 4.4.3. Analysis of Residuals and Diagnostic Plots

**Figure 20:**
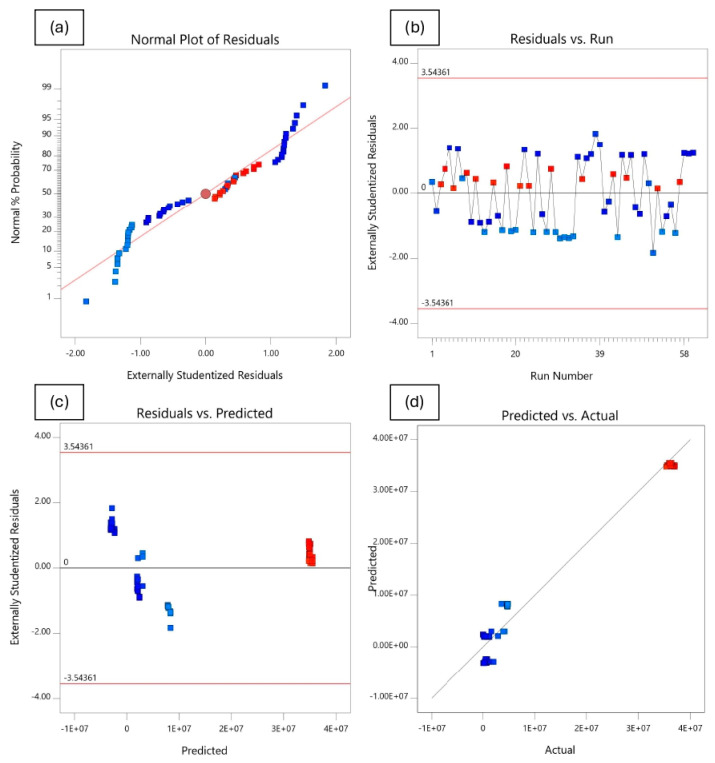
Residuals and diagnostic plots for electrical resistivity.

**Figure 21:**
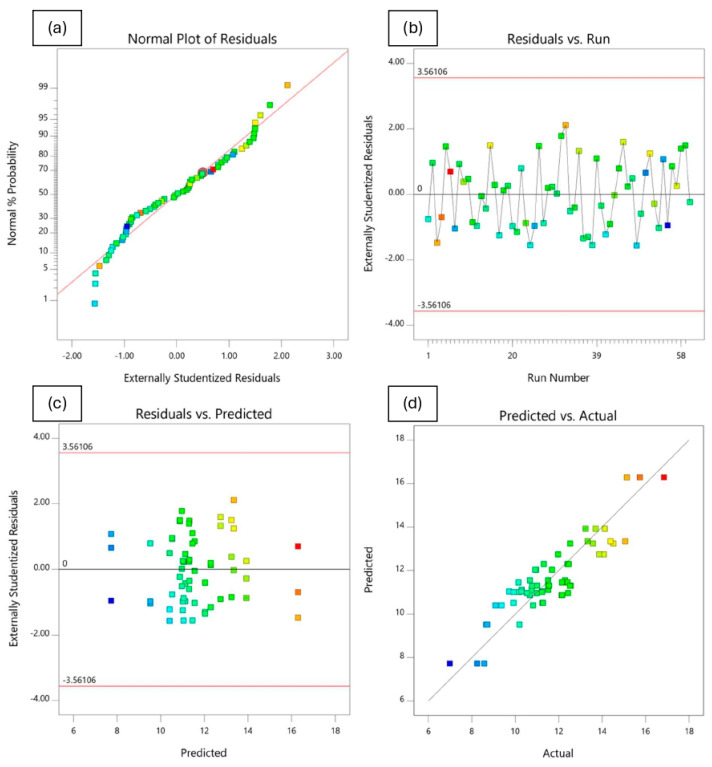
Residuals and diagnostic plots for Marshall stability.

**Figure 22:**
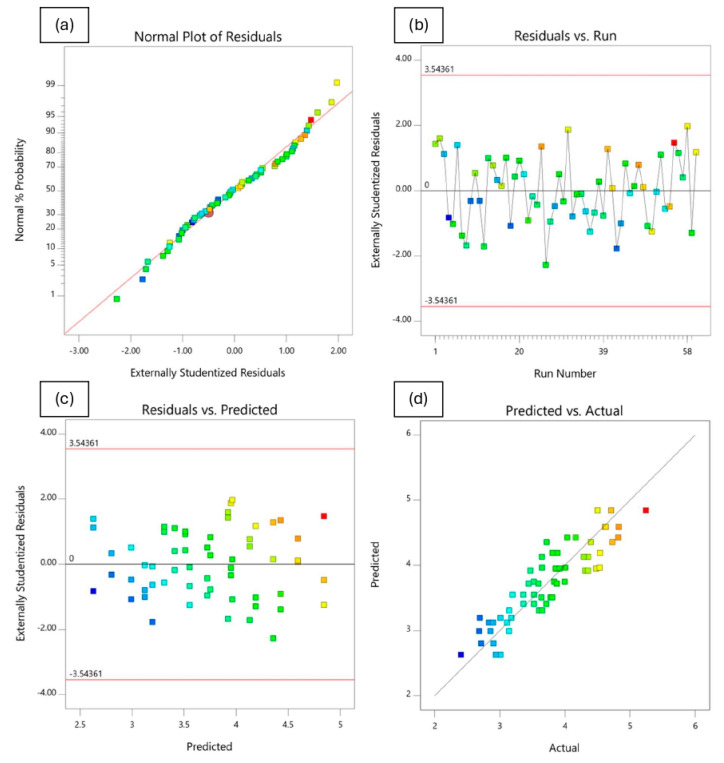
Residuals and diagnostic plots for flow.

**Figure 23:**
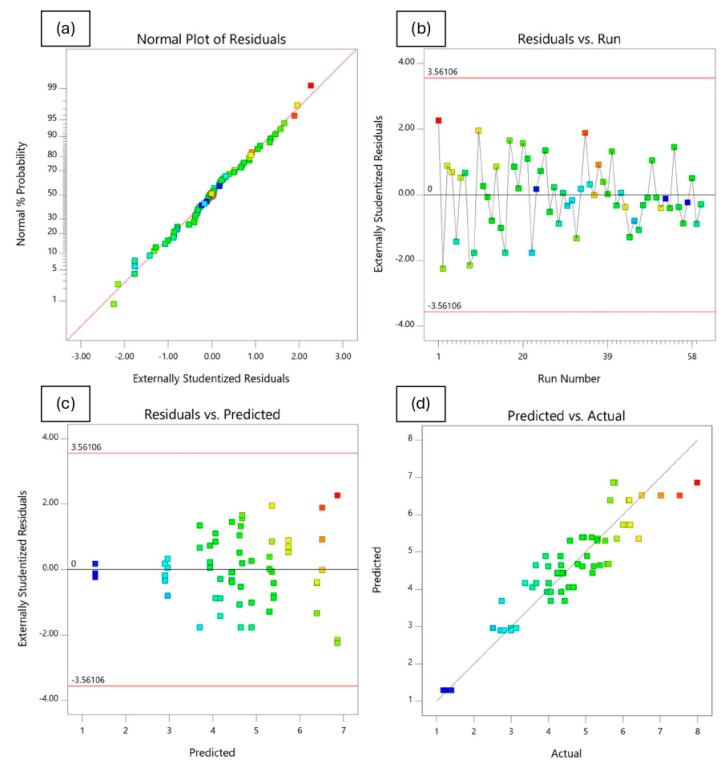
Residuals and diagnostic plots for porosity.

In addition to model validation and fit statistics, the adequacy and normal distribution of data can also be verified graphically through the diagnostic plots [66,69]. These diagnostics plots, such as normal plot of residuals, predicted vs. actual, residuals vs. predicted, and residuals vs. runs, can be used in evaluating the accuracy of the performed regression analysis. A normal probability plot is a visualization method for correlating points of data to the normal distribution. The normal probability plots of the residuals shown in Figure 20a, Figure 21a, Figure 22a and Figure 23a are almost linear, confirming the assumption that the error terms are normally distributed. The residual points on the straight line verify the response models’ normal distribution. The relationship between the normal percentage of probability and extremely studentized residuals is satisfactory since almost all the points are closely located along the straight line for the response variables. This indicates that the residual values, which is the difference between the actual and predicted responses, exhibit a normal distribution.

The residuals versus the number of run plots are used to validate the assumption that the residuals are independent of each other. Figure 20b, Figure 21b, Figure 22b and Figure 23b display the run number plots of residuals, which demonstrate a trend of data composition based on an index. It can be seen that the independent residuals are displayed in order of time, with no clear patterns. The residuals on the plot are distributed at random throughout the line of zero. This means that there are no particular errors between predicted values and the model.

Figure 20c, Figure 21c, Figure 22c and Figure 23c depict the plot of residuals according to rising predicted response values. The points are randomly distributed around the residual line of zero with no apparent pattern. It can be reported that a linear model is adequate. The graph’s red limits indicate a high level of model predictability. The data show almost no outliers since all standardized residues fall around the residual line of zero.

Figure 20d, Figure 21d, Figure 22d and Figure 23d depict graphs of predicted vs. actual values for all responses. The results show the majority of points are evenly distributed along the line of equality, indicating adequate model fit. The straight-line distributions in the plots indicated a significant correlation between predicted and measured values. In addition, plotting actual against predicted findings confirms that all predicted independent variables from mathematical models accurately match the measurements from experiments. These data demonstrate the RSM’s robustness and dependability in predicting the impact of independent variables on related dependent variables.

#### 4.4.4. Analysis of Response Factors

**Figure 24:**
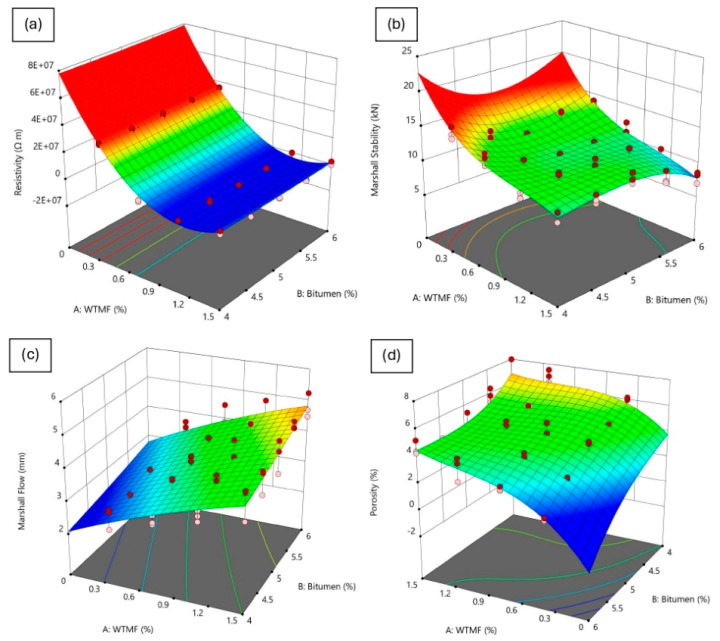
3D response surface plots for synergistic effect of response variables ((**a**) electrical resistivity, (**b**) Marshall stability, (**c**) flow, (**d**) porosity) on each input factor.

Besides the above-shown residuals and diagnostic plots, 3D response surface plots can also be used to describe the relationship between two independent variables (i.e., factors) and the corresponding response variable. These plots are a powerful way to visualize how the two independent variables influence each response variable. Figure 24 shows the 3D response surface plots of the interaction of the factors, i.e., WTMF content (X_1_) and bitumen content (X_2_), affecting the responses (resistivity, Marshall stability, flow, and porosity). The purpose of these plots is to illustrate the combined effect of two factors on each response variable which help to identify optimum mix proportions by showing trends such as peaks, valleys, and curvature, which indicate the influence of interaction effects.

Figure 24a shows the response surface plot of electrical resistivity against the input factors. The plot shows a parabolic shape, where resistivity initially decreases with increasing WTMF and then remains unchanged at higher levels, confirming the quadratic nature of the model. In Figure 24b, the response plot of Marshall stability can be seen, where a more complex interaction is observed, necessitating a cubic model. The plot exhibits multiple peaks and valleys/dips, suggesting that stability is influenced by a combination of linear, interaction, and higher-order effects. In Figure 24c, the flow response shows a smooth curvature, indicating that both X_1_ and X_2_ contribute positively to the response up to an optimal point, beyond which flow slightly decreases. For porosity, the 3D surface in Figure 24d suggests a strong interaction effect between WTMF and bitumen content. The response fluctuates, indicating that both positive and negative cubic interactions exist, which significantly impact the mix’s void structure.

#### 4.4.5. Multi-Objective Optimization and Validation

Developing a statistical analysis to ascertain the optimal content and values of WTMF and binder contents for modified HMA mixes that attain the best performance represents one of this study’s objectives. Conventional tests for establishing optimal WTMF and bitumen contents could be time and effort consuming due to their complexity and enormous quantity of data values. Accordingly, statistical evaluation and multi-objective optimization were employed throughout this study to identify the most effective amount of WTMF and binder that will result in an asphalt mixture with the best characteristics. Therefore, the optimization process aimed to maximize stability and minimize resistivity while maintaining flow and porosity within acceptable limits according to the desirability requirements. Based on the criteria set forth and the results obtained from the experiments performed, optimization was conducted using the RSM technique. Table 10 presents the optimization ranges and targeted objectives of the research parameters.

**Figure 25:**
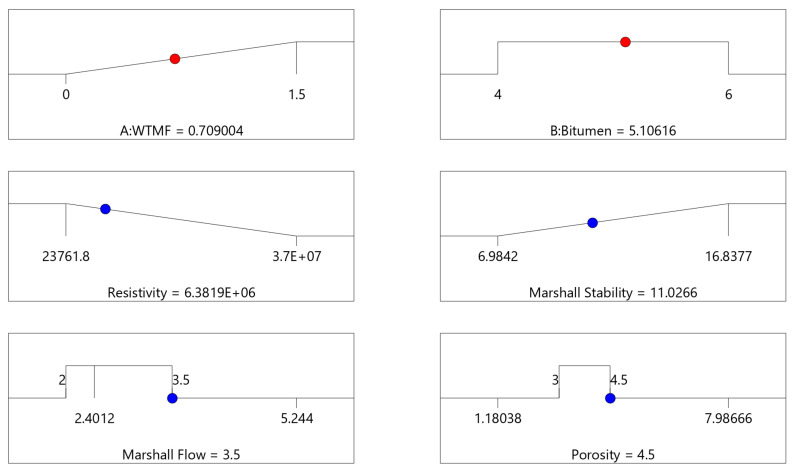
Values of RSM’s best solution for the factors (red) and responses (blue).

The RSM estimated the optimum value of the optimized variables using the mix design’s set forth desirability. RSM’s multi-objective optimization and desirability for each independent and dependent variable in the best solution are shown in Figure 25. The overlay contour plots and numerical optimization revealed that the optimal mix proportion of WTMF and bitumen content lies within the high-desirability region of the response surfaces.

The results were validated using the predicted vs. experimental values, confirming that the optimized mix meets the required performance criteria. The performance findings for the responses are given in Table 11. These findings demonstrate that the response surface methodology (RSM) approach is effective in optimizing asphalt mix properties, reducing experimental efforts while ensuring high-performance mixtures.

To validate the optimized models, further testing was conducted to evaluate the validation between the predicted model and the experimental data. Equation (12) was used to determine the percentage error, which was used to validate the predicted model and the experimental data [69].

(12) $$Error\;(\%)=\left(\frac{\left|Actual\;value-Predicted\;value\right|}{Actual\;value}\right)\times 100$$

The percent of error of the validating assessment between the optimized and experimental values is shown in Table 11. The error ratio between the RSM-predicted and experimental responses was found to be lower than 5%. This indicates that the model generated by RSM is capable of accurately and precisely predicting responses, and there is good compatibility between the findings of the RSM’s optimized solution and the experimental results.

## 5. Conclusions

The optimization of asphalt mixtures incorporating waste tyre metal fibre (WTMF) is essential to achieving a balance between electrical resistivity and mechanical performance. This study employed a multi-objective optimization approach to determine the best-performing asphalt mixtures by analysing key parameters. To achieve this, response surface methodology (RSM) was applied to predict and optimize the performance of WTMF-modified asphalt mixtures. This technique enabled the identification of optimal mix compositions that meet engineering requirements while enhancing the overall mechanical behaviour of the modified asphalt. The performance enhancement observed in WTMF-modified asphalt mixtures is primarily attributed to physical reinforcement and microstructural reorganization within the asphalt mastic. The randomly distributed fibres form a three-dimensional reinforcing network that improves stress transfer, crack-bridging capability, and resistance to deformation. No chemical interaction between WTMF and the binder is assumed, as the study focuses on mechanical and microstructural performance. The following are our conclusions in accordance with the findings of this study:

- Analysis of variance (ANOVA) was conducted to assess the statistical significance and predictive capability of the models developed for various response variables, including resistivity, Marshall stability, flow, and porosity.
- The fit statistics, including model F-values, *p*-values, R^2^ values, and adequate precision, indicate the robustness of the models.
- The results confirm that all models are statistically significant (*p*-value < 0.0001) and exhibit strong predictive capability, as indicated by high R^2^ and adjusted R^2^ values.
- The findings confirm that the models developed through response surface methodology (RSM) effectively capture the relationships between input parameters and asphalt mixture performance, ensuring reliable predictions for optimizing mix design.
- The statistical models for predicting the responses in terms of the dependent variables based on RSM analysis can be used for prediction of electrical resistivity, Marshall stability, flow, and porosity of WTMF-modified mixes based on the content level of the fibre and bitumen.

Although this study focuses on laboratory optimization of the mechanical and electrical behaviour of asphalt modified with WTMF, field performance cannot be ignored for a number of years if this is to work in the real world. The waste tyre metal fibres are mostly buried inside the asphalt mastic during service, which means that they are not in constant contact with moisture and oxygen. The surrounding protection can temper corrosion and keep things stable. But over time, the binder ages oxidatively and the environment applies stresses that may alter how the fibres interact with the binder, affecting the conductive network and influencing the overall durability of the mix. Furthering these goals of long-term performance by adding antioxidant additives could prove a beneficial complementary approach as well. Antioxidants reduce binder oxidation, reduce age-related embrittlement, and improve fatigue resistance and low-temperature cracking performance, which are all factors contributing to the durability of WTMF-infused asphalt pavements. Future research should also quantify the combined action of WTMF and antioxidant additives under aging and moisture-conditioning scenarios to better quantify the long-term mechanical, electrical, and durability performance in field-like conditions. Moreover, although this study focused on the performance optimization of asphalt using WTMF, the underlying principles of enhancement, including fibre-induced reinforcement and improved microstructural organization, are likely applicable to other polymer- or fibre-modified asphalt systems. The actual performance improvements, however, will depend on the specific nature and content of the additives. Therefore, the trends and findings reported here can serve as a benchmark for future studies involving different modified asphalt mixtures.

## Figures and Tables

**Table 1:** Physical properties of aggregates and bitumen.

Property	Aggregates	Bitumen
Penetration	-	63 dmm
Ductility	-	135 cm
Softening point	-	49.6 °C
Specific gravity	2.60	1.03
Water absorption	4%	-
Los Angeles abrasion	21.54%	-
Gradation	JKR’s AC-14	-
Flakiness	21.4%	-
Elongation	22.7%	-
Crushing value	24%	-

**Table 2:** Description of factors and responses.

Type	Parameter	Unit	Code
Factor	WTMF	%	X_1_
Factor	Bitumen	%	X_2_
Response	Stability	kN	Y_1_
Response	Flow	mm	Y_2_
Response	Electrical resistivity	Ωm	Y_3_
Response	Porosity	%	Y_4_

**Table 3:** Experimental factors and their coded levels for RSM.

Factor	Unit	Symbol	Coded Values					Limits
			−α	−1	0	+1	+α	
WTMF content	%	X_1_	0	0.375	0.75	1.125	1.5	0, 0.375, 0.750, 1.125, 1.500
Bitumen content	%	X_2_	4	4.5	5	5.5	6	4.0, 4.5, 5.0, 5.5, 6.0

**Table 4:** EDS quantitative analysis.

Element	Atomic Percent	Weight Percent
Carbon	57.32	28.99
Copper	2.70	7.23
Iron	21.97	51.65
Oxygen	18.01	12.13
Total	100	100

**Table 5:** XRD peak parameters.

Peak	Position (°2θ)	Net Height (Counts)	FWHM (°)
1	44.785	333.1	0.836
2	65.689	115.9	0.832
3	82.261	205.0	1.337

**Table 6:** Crystallite size calculation using Scherrer’s equation.

Peak	Position (°2θ)	FWHM (°)	FWHM (rad)	Crystallite Size (nm)
1	44.785	0.836	0.0146	102.78 nm
2	65.689	0.832	0.0145	113.65 nm
3	82.261	1.337	0.0233	78.89 nm

**Table 7:** Marshall parameters and volumetrics with different percentages of WTMF and OBC.

Series	OBC (%)	Bulk Specific Gravity (G_mb_)	Porosity (%)	Voids in Mineral Aggregates (VMA) (%)	Voids Filled with Bitumen (VFB) (%)	Stability (kN)	Flow (mm)
Control	4.6	2.3316	4.03	14.45	72.23	15.38	2.21
Series A	4.9	2.3154	4.30	15.31	71.98	12.89	2.87
Series B	5.0	2.3111	4.47	15.47	71.14	12.69	3.84
Series C	4.9	2.3033	4.79	15.75	69.60	12.33	3.94
Series D	4.5	2.3091	5.10	15.18	66.48	12.10	4.10

**Table 8:** Resistivity data for each mix series.

Series	WTMF Content (%)	Resistance (MΩ)	Resistivity (Ωm)
Control	0	∞	∞
Series A	0.375	304.51	$3.62\times 10^{7}$
Series B	0.75	40.40	$4.73\times 10^{6}$
Series C	1.125	5.34	$6.18\times 10^{5}$
Series D	1.5	11.03	$1.26\times 10^{6}$

**Table 9:** ANOVA’s fit statistics for each response variable.

Metric	Response Variables			
	Resistivity (Ωm)	Stability (kN)	Flow (mm)	Porosity (%)
Model F-value	326.51	23.10	45.31	30.16
Model *p*-value	<0.0001	<0.0001	<0.0001	<0.0001
Mean	1.07 $\times 10^{7}$	11.63	3.72	4.61
R^2^	0.96	0.80	0.81	0.84
Adjusted R^2^	0.95	0.77	0.79	0.82
Predicted R^2^	0.96	0.72	0.7578	0.79
Adequate precision	43.60	22.72	23.00	22.45
Remarks	Model is significant	Model is significant	Model is significant	Model is significant
Final suggested model	Quadratic	Cubic	Quadratic	Cubic

**Table 10:** Target criteria for optimizing the mixture design.

Factor/Response	Criterion	Lower Limit	Upper Limit
WTMF content	Is in range	0	1.50%
Binder content	Is in range	4%	6%
Electrical resistivity	Minimize	0	∞
Marshall stability	Maximize	12 kN	16 kN
Flow	Is in range	2 mm	4 mm
Porosity	Is in range	3%	5%

**Table 11:** Multi-objective optimization and experimental validation of predicted results.

Factor/Response	Unit	Predicted Result	Experimental Result	Error (%)
WTMF	%	0.71	-	-
Bitumen	%	5.11	-	-
Electrical resistivity	Ωm	6.38 × 10^6^	6.12 × 10^6^	4.3
Marshall stability	kN	11.03	10.65	3.6
Flow	mm	3.5	3.62	3.3
Porosity	%	4.5	4.61	2.4

## Data Availability

The original contributions presented in this study are included in the article. Further inquiries can be directed to the corresponding authors.
